# Indications and techniques of corneal transplants performed in one center in Southern Poland, in the years 2001–2020

**DOI:** 10.1371/journal.pone.0276084

**Published:** 2022-11-18

**Authors:** Dominika Szkodny, Ewa Wróblewska-Czajka, Adam Wylęgała, Edward Wylęgała

**Affiliations:** 1 Chair and Clinical Department of Ophthalmology Faculty of Medical Sciences in Zabrze Medical University of Silesia in Katowice, Katowice, Poland; 2 Department of Ophthalmology, District Railway Hospital in Katowice, Katowice, Poland; Singapore Eye Research Institute, SINGAPORE

## Abstract

**Purpose:**

The study aimed to evaluate changes over a period of time in techniques and indications for corneal transplantation in a single center in Poland.

**Methods:**

Retrospective analysis of corneal transplants performed at the Ophthalmology Department of the District Railway Hospital in Katowice in 2001–2020. Data on indications and surgical technique were collected.

**Results:**

A total of 3021 corneal transplantations were performed in the years 2000–2020. The most common technique was penetrating keratoplasty (69,54%), then lamellar grafts—both anterior and posterior (19,63%), and limbal grafts (10,82%). Only in 2007 the number of lamellar keratoplasties exceeded the number of penetrating grafts and accounted for 108 (54,55%), including 85 (42,93%) of Descemet Stripping Automated Endothelial Keratoplasty. The only noticeable trend in changing techniques was a decrease in the number of performed deep anterior lamellar keratoplasties (r 2 = -0.21) over the last seven years. Main indications included bullous keratopathy (23,22%), following keratoconus (18,59%), leukoma (14,67%), keratitis and perforation (14,3%), Fuchs dystrophy (11,4%), and regrafts (7,22%). Leukoma and bullous keratopathy as an indications for corneal transplant have been declining over the years (r2 = 0.60 and r2 = 0.30 respectively). Consecutively, indications such as fuchs dystrophy, regraft and keratitis perforation have increased (r2 = 0.05, r2 = 0.50, r2 = 0.33)

**Conclusions:**

The number of keratoplasties gradually increased from 2001 to 2020. It could be possible that the development and spread of new therapeutic alternatives, like corneal cross-linking and scleral lenses, have contributed to the decrease in deep anterior lamellar keratoplasty performed. There was an increasing trend in the percentage of penetrating keratoplasties for failed grafts, Fuchs dystrophy and infectious keratitis.

## Introduction

Penetrating keratoplasty (PKP), performed for the first time by Eduard Zirm in Olmütz in 1905, is still one of the oldest and most commonly practiced and most effective transplantation worldwide [1–4]. Despite PKP remains as the gold standard in various indications, in the last decades, other corneal transplantation techniques–anterior and posterior lamellar keratoplasties–have been developed, which have shown to have several advantages. In several recent studies endothelial pathologies (bullous keratopathy, Fuchs endothelial dystrophy and regraft) have become the main indications for corneal transplantation, and this shift stimulated the need to search for less invasive procedures [2, 5–8]. Due to its multiple advantages over the PKP, resulting from the possibility of selective replacement of corneal layers, lamellar keratoplasty techniques have dominated PKP in specific indications [9]. Its benefits include faster visual rehabilitation, better visual acuity, less invasive, smaller refractive error and significantly lower risk of immune rejection [10, 11].

Indications and techniques for corneal transplant vary by geographic regions, which is influenced not only by the prevalence of certain corneal diseases but also by the state of health service and economy [1, 12]. The number of performed lamellar keratoplasties depends on various factors regarding tissue availability, early diagnosis of corneal diseases, dominant indications for keratoplasty, and advances in other ophthalmic procedures like cataract surgery and cross-linking [7, 13]. The experience of the surgeon is also important, as the fear of damaging the graft during preparation or of failure of the operation due to a more complicated technique may affect the more willing choice of penetration technique. Poland belongs to the countries where both the number of performed lamellar keratoplasties and the total number of transplants remain at a low level, mainly due to donor shortage [14]. The purpose of the current study was to identify longitudinal trends in indications and techniques of corneal transplants performed in District Railway Hospital in Katowice, Poland, in the years 2000–2020 and compare outcomes to global reports.

## Materials and methods

This retrospective study included all patients who underwent corneal transplantation between January 2001 and December 2020 at the Department of Ophthalmology of District Railway Hospital, Katowice, Poland. The present research adhered to the tenets of the Declaration of Helsinki. The ethics committee of Medical University of Silesia has stated that their approval for this study and participant consent is not required. ‘Patients’ data collected includes age, sex, date of surgery, indication for surgery, surgeon, corneal transplant technique, and preoperative visual acuity. Data from 2001–2015 were collected from the paper charts and 2016–2020 from the electronic register. The surgical procedures were categorized into the following groups: penetrating keratoplasty (PKP, triple procedure), deep anterior keratoplasty (DALK), posterior lamellar keratoplasty, including Descemet stripping automated endothelial keratoplasty (DSAEK) and Descemet Membrane Endothelial Keratoplasty (DMEK), and limbal graft (keratolimbal allograft -KLAL, Conjunctival *Limbal* Autograft -CLAU, Simple Limbal Epithelial Transplantation- SLET, and Cultivated Limbal Epithelial Transplantation- CLET).

Indications in our study were divided into the following groups: bullous keratopathy, regraft, keratoconus, leukoma, ’Fuchs’ dystrophy, limbal stem cell deficiency (lscd), and keratitis with perforation. In patients with leukoma were also those after corneal injury with scar obscuring the axis of vision. Cases of bullous keratopathy with Fuchs dystrophy were classified as ’Fuchs’ dystrophy. After keratoplasty in our Department standardized treatment includes two topical antibiotics–levofloxacin, tobramycin, Dexamethason 0,1%, moisturizing drops every two hours and systemic: ciprofloxacin 500 mg 2 x per day and methylprednisolone 16 mg. Sutures are not removed until they are torn off or their significant influence on keratometry is suspected. Corneoscleral rim for the donors were used for transplant. The primary outcome were trend in indications and techniques for corneal transplant in our region over the last 20 years. Data were collected in 2021.

Pearson’s correlation coefficient and linear regression were used to determine the trend of changes in the number and types of treatments over the years. Indications, number, and techniques of keratoplasties were analyzed and compared between each year (using chi-square test),. Data were visualized on scatter plots with plotted regression lines and 95% confidence intervals. The Student’s T-test with Brown Forsyth’s test was used to compare the age of the patients to assess the homogeneity of variance. The analysis was performed using the RStudio (RStudio Team, 2016, Boston, Massachusetts) package and the ggplot library. A p-value <0.05 was considered statistically significant.

## Results

A total of 3021 grafting procedures were performed from January 2001 through December 2020 at the Opthalmology Department, District Railway Hospital in Katowice, including 2101 (69,54%) PKP, 593 lamellar grafts (19,63%), and 327 (10,82%) limbal grafts. Mean recipient age was 61.22 ± 18,48. The majority of graft recipients were female 1572 (52,04%).

**Fig 1:**
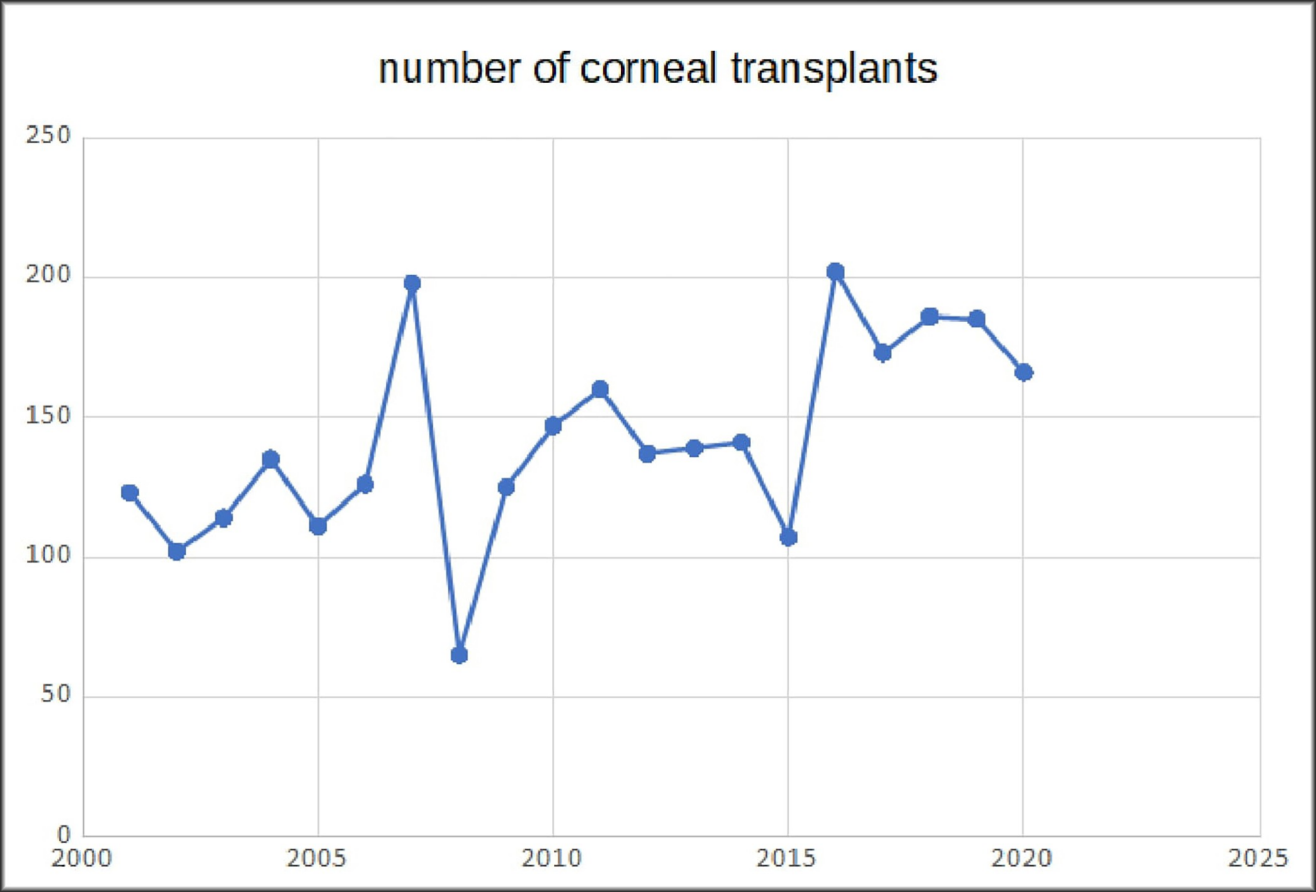
Number of performed corneal grafts in analyzed period.

**Fig 2:**
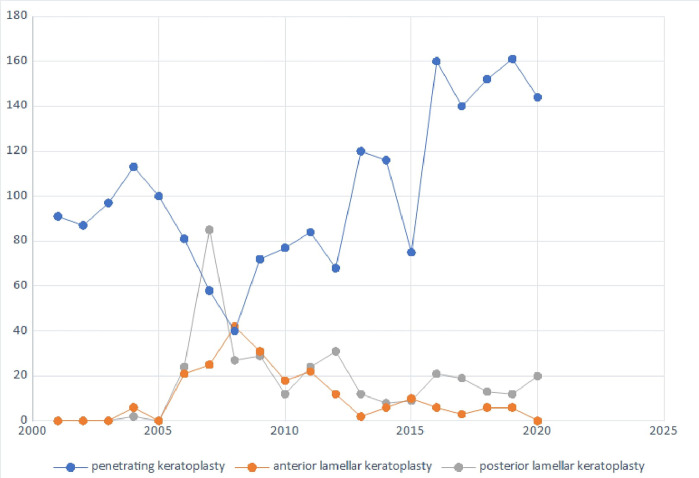
Number of different keratoplasty techniques in analyzed period.

Over 20 years, a slight upward trend in the number of transplants performed has been observed, with two peaks in 2007 (198 corneal grafts) and 2016 (202 corneal grafts) (Fig 1). Between 2006 and 2012, lamellar keratoplasties have slightly increased and after this time decreased reaching plateau by 2020. Also, in 2007 the largest number of lamellar keratoplasties was performed, accounting for 55,55% of all grafts performed this year. In this period, the number of posterior lamellar keratoplasties was also the highest, containing 85 DSAEKs (Fig 2). This increase was related to the introduction in our department ultrathin DSAEK procedure. However, this trend was not observed in the following years due to the lower number of corneal grafts performed in total. From 2006–2011, the highest number of anterior lamellar keratoplasties was recorded with a decreasing tendency in the next years. The percentage of lamellar keratoplasties remained low throughout the observed period, apart from the peak in 2007.

Limbal grafts accounted for a significant part of the total number of corneal grafts, which was associated with the introduction of the CLET procedure in our department.

**Fig 3:**
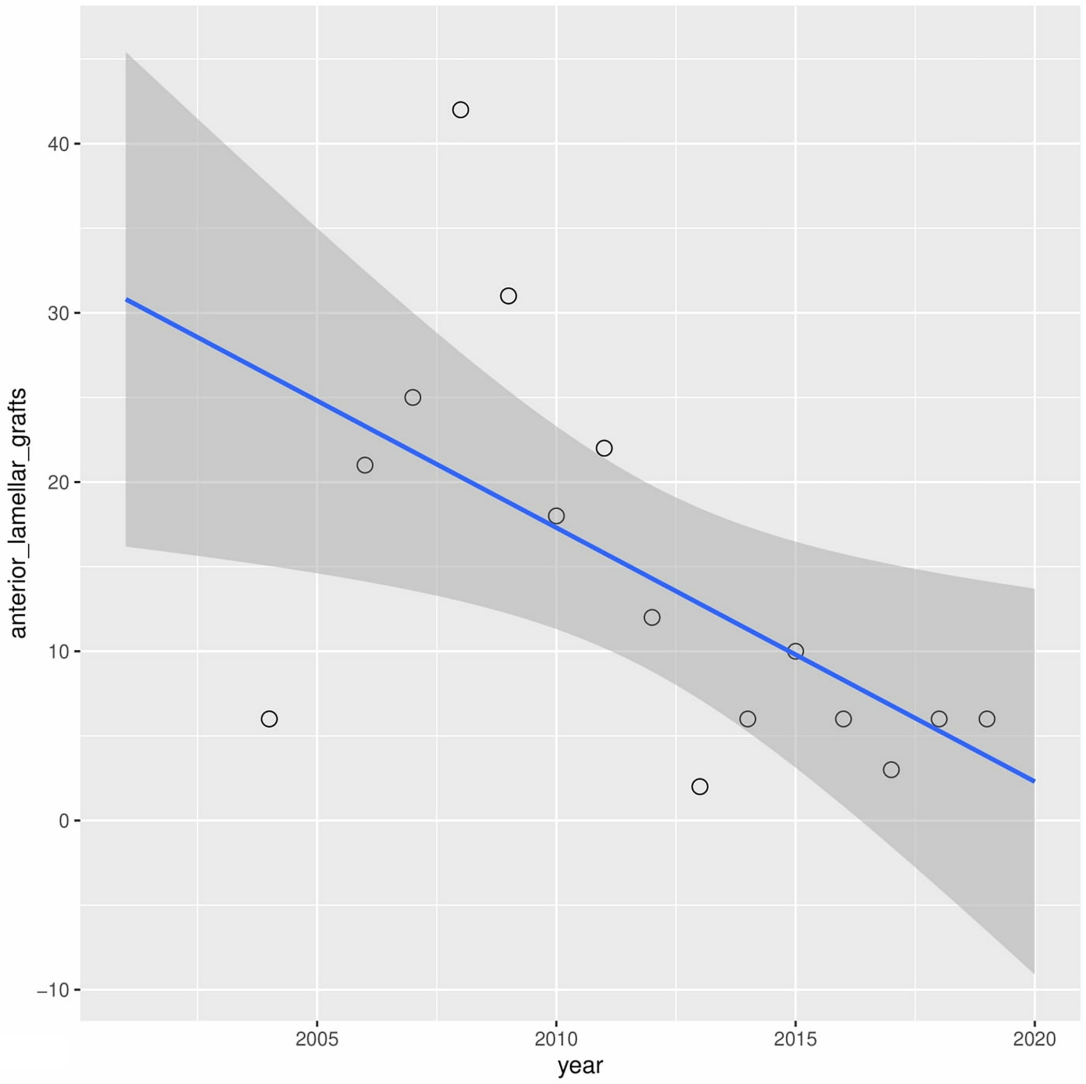
Presents univariate linear regression analysis of the annual incidence for anterior lamellar keratoplasty and penetrating keratoplasty.

**Fig 4:**
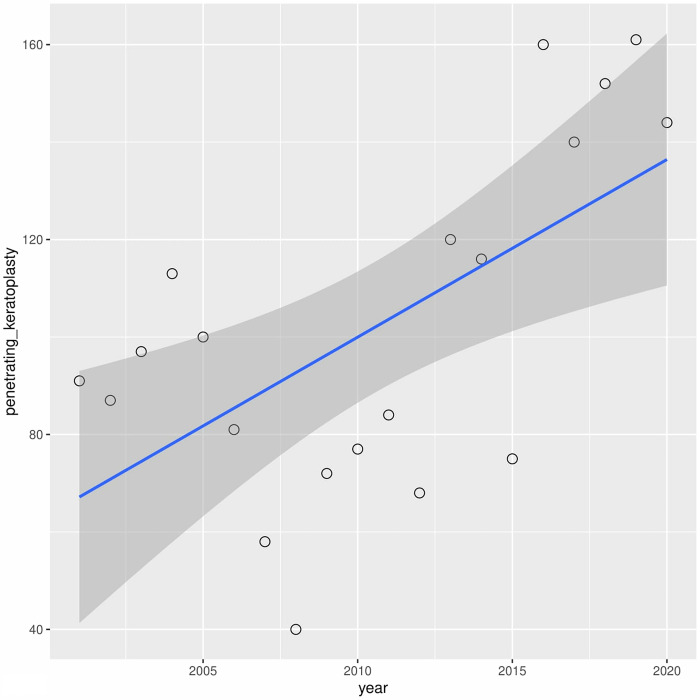
Presents univariate linear regression analysis of the annual incidence for anterior lamellar keratoplasty and penetrating keratoplasty.

Univariate linear regression analysis of the annual incidence for each type of procedure revealed a significant decrease in anterior lamellar keratoplasty (r2 = 0.21) and a significant increase in penetrating keratoplasty (r2 = 0.35) (Figs 3 and 4). Other surgical procedures have not revealed significant fluctuations.

**Fig 5:**
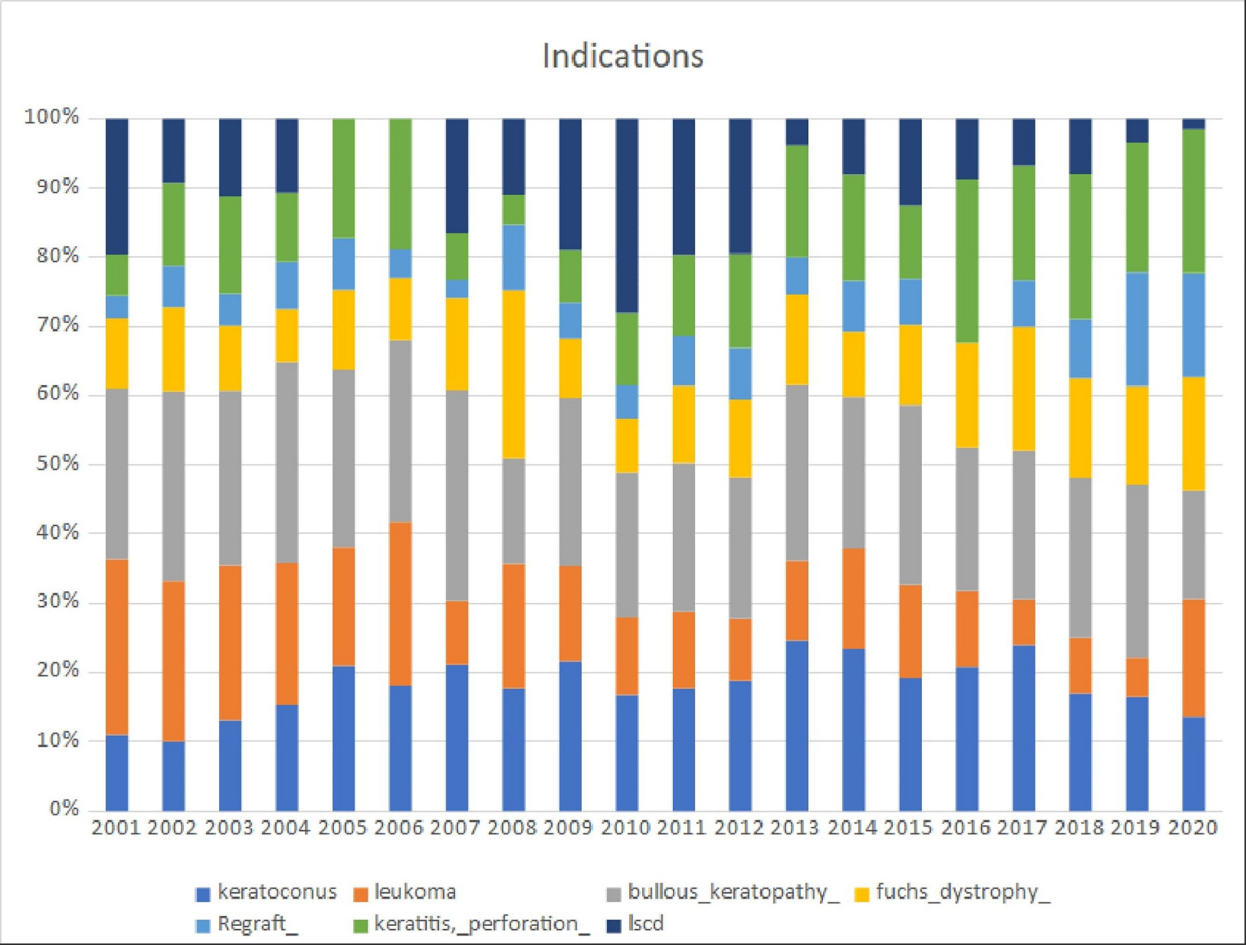
Structure of indications for keratoplasty in alayzed period.

The most common indication over the last 20 years was bullous keratopathy (23,22%), following keratoconus (18,59%), leukoma (14,67%), keratitis and peroforation (14,3%), Fuchs dystrophy (11,4%) and regrafts (7,22%) (Fig 5).

**Fig 6:**
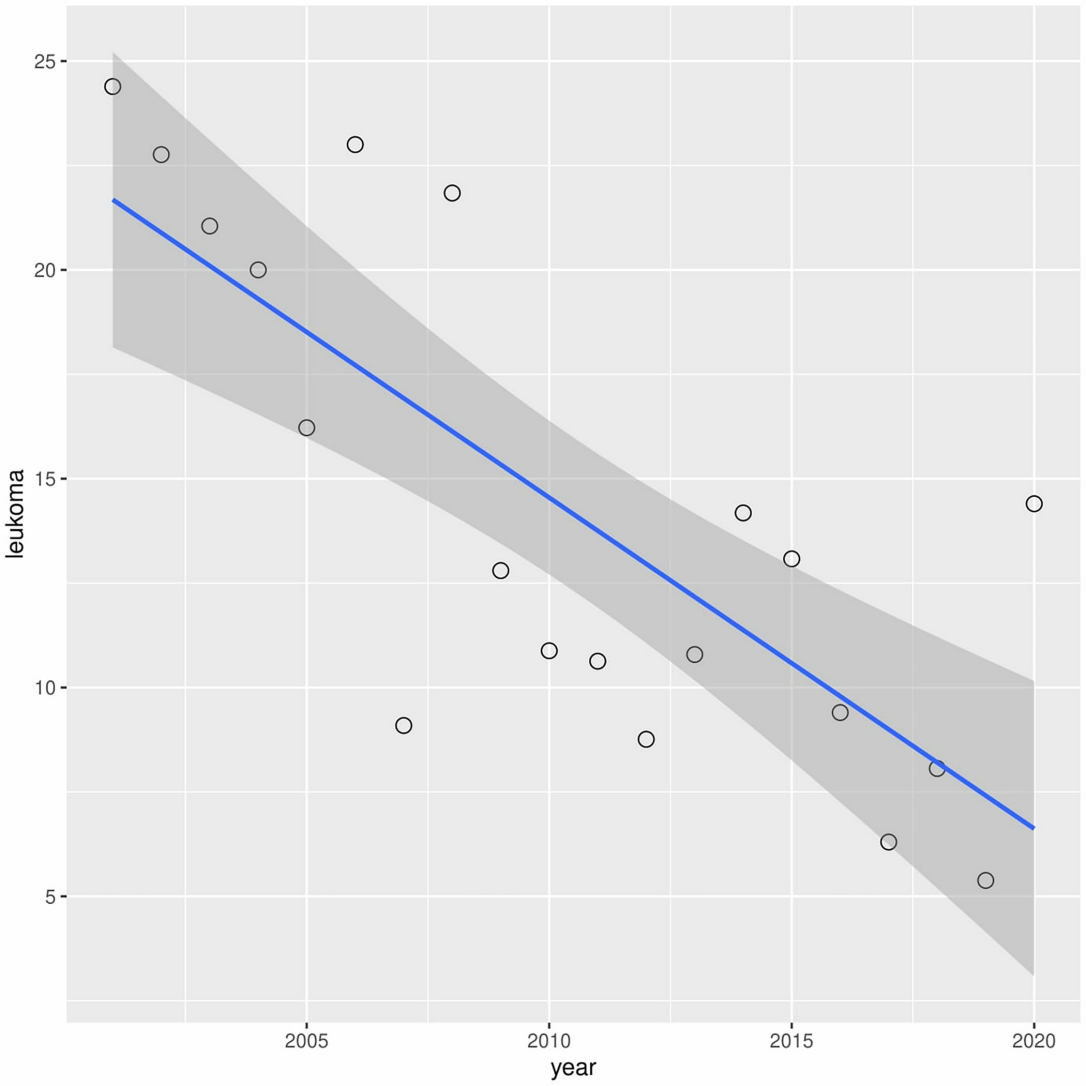
Presents univariate linear regression analysis of the annual incidence for different indications.

**Fig 7:**
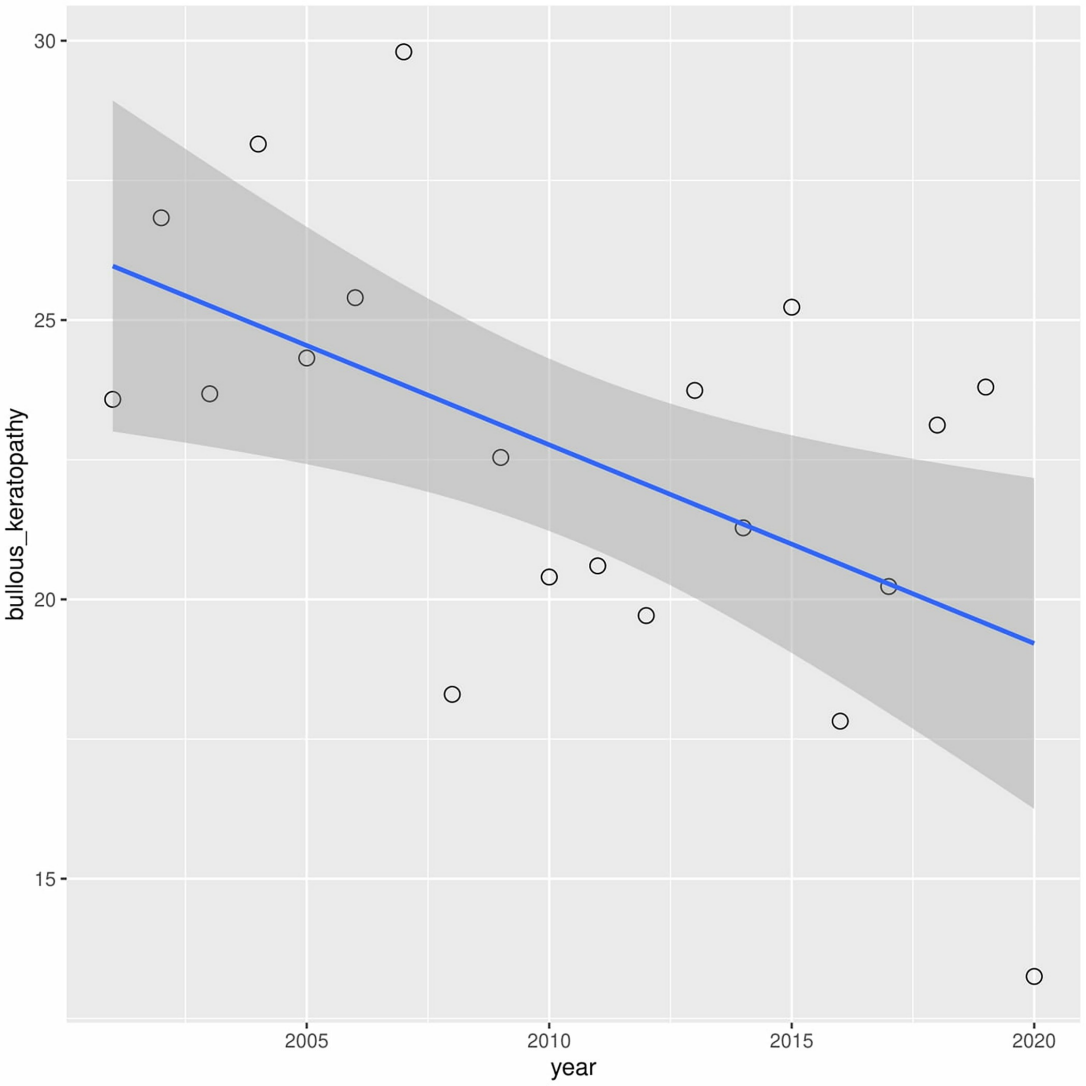
Presents univariate linear regression analysis of the annual incidence for different indications.

**Fig 8:**
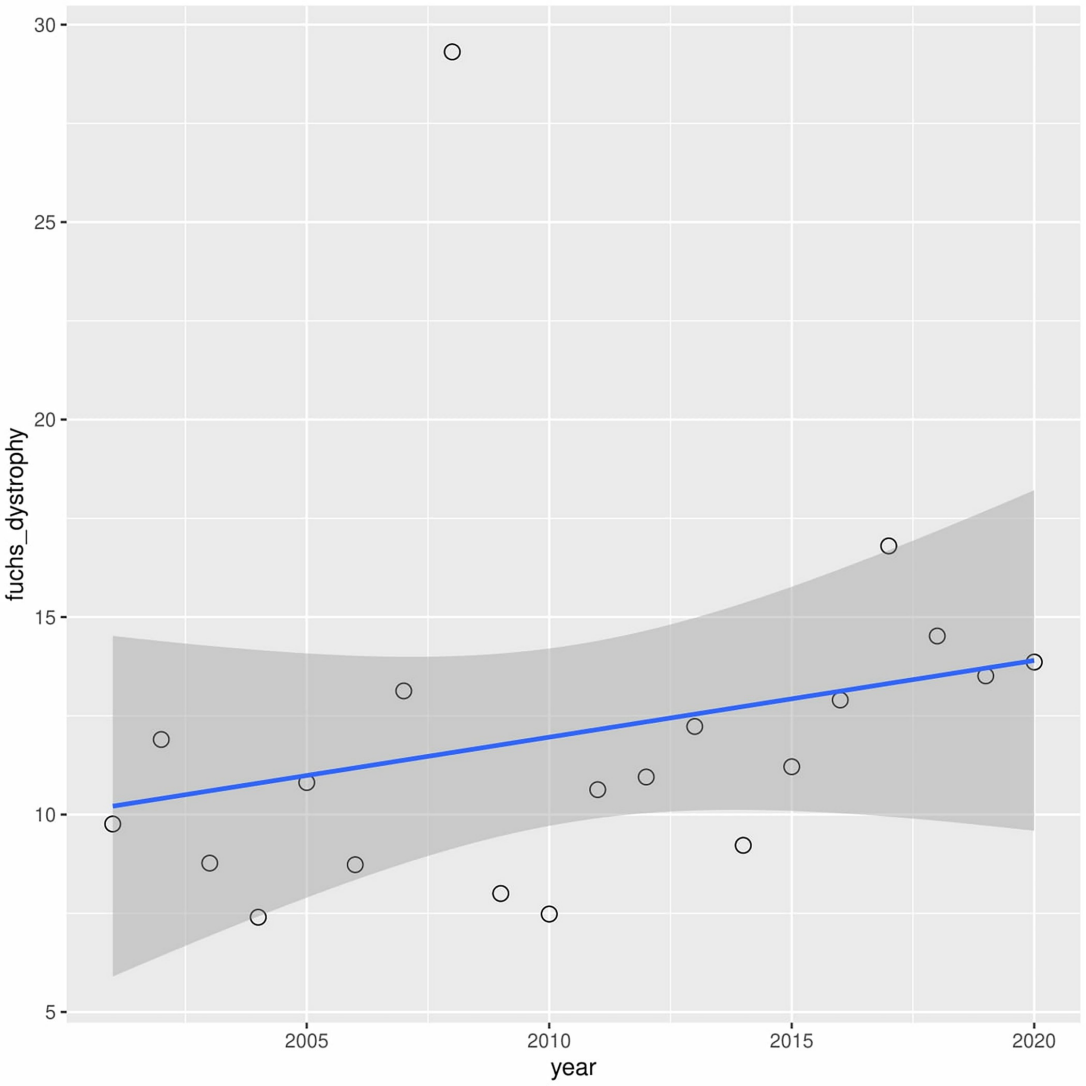
Presents univariate linear regression analysis of the annual incidence for different indications.

**Fig 9:**
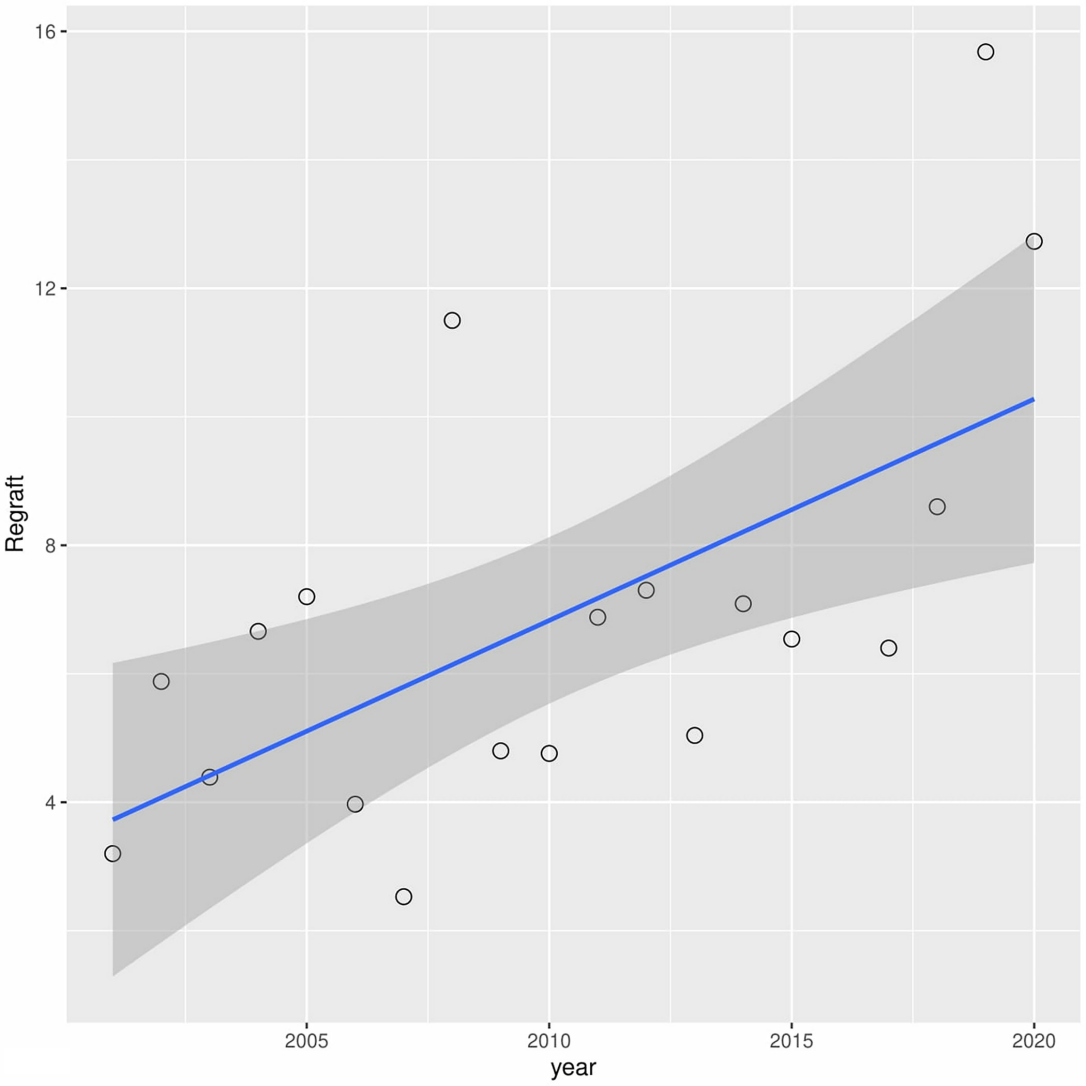
Presents univariate linear regression analysis of the annual incidence for different indications.

**Fig 10:**
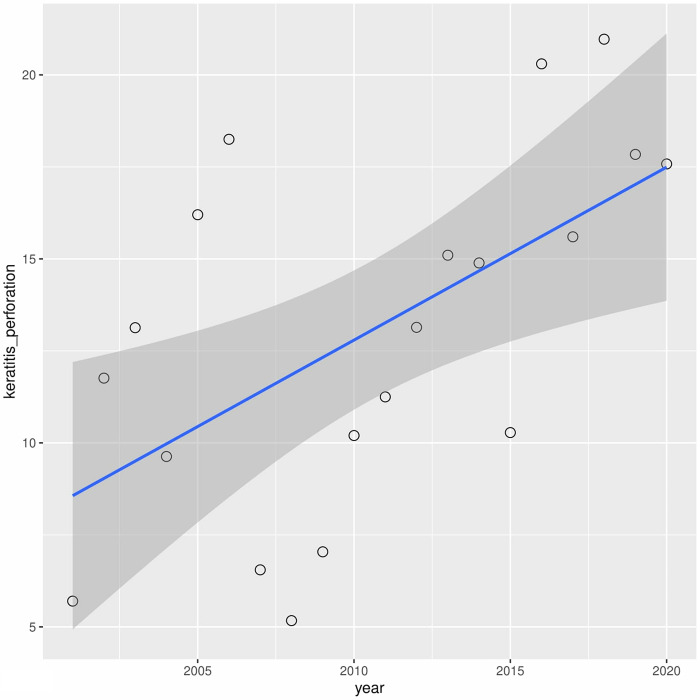
Presents univariate linear regression analysis of the annual incidence for different indications.

A downward trend in leukoma has been observed over the years (r2 = 0.60) (Fig 6). A similar situation occurred with bullous keratopathy (r2 = 0.30) (Fig 7). In turn, in cases of fuchs dystrophy, regraft and keratitis perforation number of performed keratoplasties have increased (r2 = 0.05, r2 = 0.50, r2 = 0.33 respectively) (Figs 8–10).

## Discussion

This retrospective study provides an overview of absolute numbers of corneal transplantations in a single center. Further, the linear regression was performed to show the trends in the various types of and indications for corneal transplantation.

In a survey to surgeons and eye banks, in 2012, according to global number of procedures performed, Fuchs’endothelial dystrophy, Gain et al. found to be the most common cause of corneal transplantation (39% of the cases). However, the indications for keratoplasty varies according to the geographical location, with the prevalence of anterior corneal pathologies in the developing world in contrast to the dominance of endothelial diseases reported in developed countries, like the US, where the endothelial keratoplasties are becoming predominant [15]. Gain P, Jullienne R, He Z, Aldossary M, Acquart S, Cognasse F, Thuret G (2016) Global survey of corneal transplantation and eye banking. JAMA Ophthalmol 134:167–173. However in Asia, the most common prevalent transplantation was PKP, with infectious keratitis being the leading cause of transplants [11, 16]⁠⁠.

In our study bullous keratopathy was the main indication for corneal transplant over the recent years, similarly to reports from Hungary, Greece, Japan, Spain, North and South America [10, 17]. However, a decreasing trend has been observed, probably related to the improvement of cataract surgery techniques, and the almost universal use of ophthalmic viscosurgical devices [5].

On another note, despite an observed decreasing tendency, keratoconus remains the main indication of PKP in some series from Germany, Australia, New Zealand, Middle East, Africa, South America, UK and Canada [11, 17–19]. In some areas of Asia keratoconus is consistently the first indication, probably related with the very high prevalence of this disease in some of these countries, and that the contact lenses may be less tolerated in countries with a drier climate.

On the other hand, it has been reported, that a declining trend in keratoplasties due to keratoconus in some countries might be associated the availability of other surgical procedures like corneal crosslinking [5, 18, 20, 21].

The wider use of corneal topography helped to detect keratoconus earlier and allowed to inhibit disease progression by collagen cross-linking [22, 23]. Furthermore, the development of scleral contact lenses technology enabled many patients to achieve satisfying visual acuity without necessity of corneal transplant. Finally, conflicting reports regarding the visual outcomes of PK and DALK may alter corneal surgeons’ practice, limiting the performance of anterior lamellar keratoplasties, because the interface opacification is thought to limit the visual outcome in DALK [10, 24, 25].⁠

Nevertheless, anterior lamellar keratoplasty still has an advantage over PKP in terms of complications–lower risk of transplant rejection or secondary glaucoma. Further, new techniques using femtosecond laser may improve postoperative visual acuity in those patients [26–29]. ⁠Decrease in percentage of DALK was observed in our study, although keratoconus remained a second most frequent indication through all analyzed years. It may be associated with a large number of keratoconus patients referred to our hospital as our department is one of the few performing cross-linking free of charge. Also, it is described in the literature that the gap between introducing cross-linking and a decline in keratoplasty performed due to keratoconus is estimated at 10 years. Therefore, it is very likely that it will be observed in our hospital in next years.

Global indications for corneal transplants seem to be influenced by multiple factors. The reports mention, the occurrence of certain corneal pathologies, socioeconomic factors and the access to novel tissue storage and surgical techniques, society attitudes to eye donation and eye banking system [12].

The growing proportion of regrafts presented in our study is a consequence of an aging patients, advanced stages of primary diseases, and limitation of graft survival. This observation has also been described in other studies [13].

Flockerzi et al. reported decreasing tendency in the proportion of PKPs in Germany, from 96.0% in 2006 to 40.1% in 2016, and an increase of posterior lamellar keratoplasties from 14% in 2006 to 57% in 2016 [2]. This trend has been observed in other developed countries, among others the USA, UK, Italy, Japan, Ireland [6, 10, 30].⁠⁠⁠⁠

The number of lamellar keratoplasties performed in our hospital is highly unsatisfactory. This tendency is consistent with other ophthalmology departments in Poland and has its source mainly in donor tissue shortage [11]. Following Mathews PM et al. Report, it is still a global problem -approximately 90% of all corneal transplantation surgeries performed in 95 reporting countries are PK, regardless of surgical indication and one-third of countries performed no lamellar keratoplasties [1].

Diverse factors may limit the shift towards endothelial techniques, including the issue of preparing the donor tissue, which may lead to surgeon’s concern about a lamellar graft preparation failure and, consequently, graft waste. In addition, posterior lamellar transplants must be done early, when the corneal stroma has not yet suffered fibrosis due to being swollen for too long, and with inflammatory episodes that lead to fibrosis, and that make PKP necessary, and donor tissue shortage in some countries therefore causes many patients who initially might be candidates for an endothelial transplant, ultimately will require a penetrating one. On another note, there are also the technical challenges when inserting the lamellar donor tissue, surgical step that requires very specific training [31, 32]. Hence some eye banks have started providing precut and pre-stripped donor corneal tissue for DMEK [33]. Other banks offer preloaded tissues, further reducing the risk of failure and costs of the procedure [34].

Furthermore, lack of tissue availability reduces access to corneal transplants for patients with better visual acuity, leading to prolonged waiting time [33]. It is commonly known and described in different studies that the outcome of a DMEK surgery in endothelial disorders is limited by the long waiting time of patients for the surgery [6].⁠ Preoperative visual acuity values below 20/100 result in significantly poorer visual outcomes after posterior lamellar keratoplasty [35].⁠ In the study performed by Röck T et al., DMEK was performed based on the patient’s complaint, even if the visual acuity was 0.6 or better [6]. Moreover, excellent visual outcomes have been reported after DMEK procedure, reaching a BCVA of >20/25 (0.8) in 82% of examined patients [36]. Development of microsurgical techniques and the tremendous success of the DMEK has allowed many patients with endothelial diseases to perform keratoplasties at an earlier stage than before [4, 6, 19]. However, in our study, none of the patients had preoperative BCVA 0.6 or better, and only one had 0.5, prioritizing those with poorer visual acuity.

⁠PKP presents several drawbacks, including postoperative astigmatism, corneal wound dehiscence, allograft rejection, and early and late endothelial failure [37].

The most common reasons for the failure of corneal transplants in our hospital were endothelial failure, perforation and bacterial keratitis. All of them are influenced mainly by primary indication, therefore careful qualification for transplant and pre and postoperative recommendations, like proper antibiotic therapy in keratitis are crucial to increase the chances of success. However, indications such as corneal perforation, limbal stem cell deficiency or previous corneal graft failures are known to have high failure rates worldwide and still stand therapeutic challenge. Therefore, for patients who are not candidates for keratoplasty, Keratoprosthesis may be considered.

The relatively low percentage of posterior lamellar keratoplasty grafts did not bring significant shifts in corneal indications.

A lack of corneal tissue and demographic change due to population aging and the increasing need for posterior lamellar procedures creates a dire necessity for improving tissue procurement. We perform Cultivated Limbal Epithelial Transplantation (CLET) at our hospital, however, we do not have a possibility to harvest corneal cells in the lab.

PKP presents several drawbacks, including postoperative astigmatism, corneal wound dehiscence, allograft rejection, and early and late endothelial failure [31]. Therefore, it is substantial to strive to perform more lamellar keratoplasties.

Our study revealed, that despite of increasing percentage of lamellar techniques in developed countries, in our region it still remain in the minority, therefore, it could not influence the indications. Main reason for this situation is donor shortage, which on the one hand, it does not allow for sufficiently early qualification for lamellar transplant, on the other heightens surgeons’ concerns about corneal graft damage and prompts them to use penetrating techniques.

## ⁠Limitation

Our study is limited by its retrospective nature with potential for bias and heterogeneity in available data. Further, the study was done in a tertiary center with a high rate of referrals. Moreover, some of the data from earlier years were missing; however, considering many analyzed cases, these shortages were irrelevant.

To conclude, over the last 20 years, we have not observed considerable shifts in indications and techniques of corneal transplantation in our hospital, contrary to other developed countries. However, this is likely the result of the limited availability of corneal tissue for transplantation. Furthermore, a major effort should be undertaken to cover this gap regarding a potential vast donor pool and many patients waiting for keratoplasty in Poland. Therefore a close collaboration among government, ophthalmologists, public health officials, and patients is necessary.

## Supporting information

S1 Raw images (PDF)
