# Supplementary material for: Indications and techniques of corneal transplants performed in one center in Southern Poland, in the years 2001–2020
Source: PLoS One. 2022 Nov 18;17(11):e0276084. doi: 10.1371/journal.pone.0276084 (PMC9674162; doi:10.1371/journal.pone.0276084)
Supplement: S1 Raw images — (PDF) [file pone.0276084.s001.pdf]

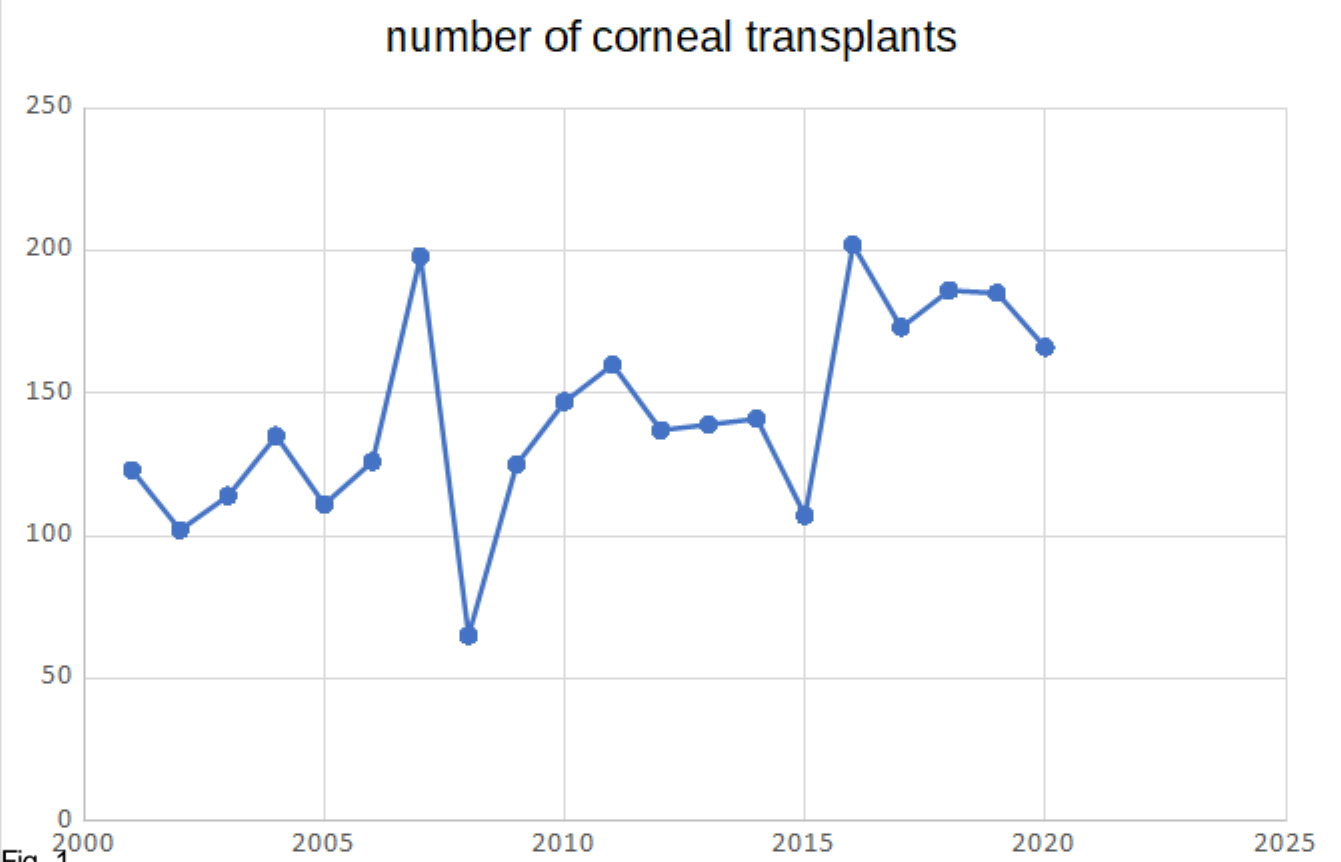
number of corneal transplants
250
200
150
100
50
0
2000
2005
2010
2015
2020
2025
Fig. 1

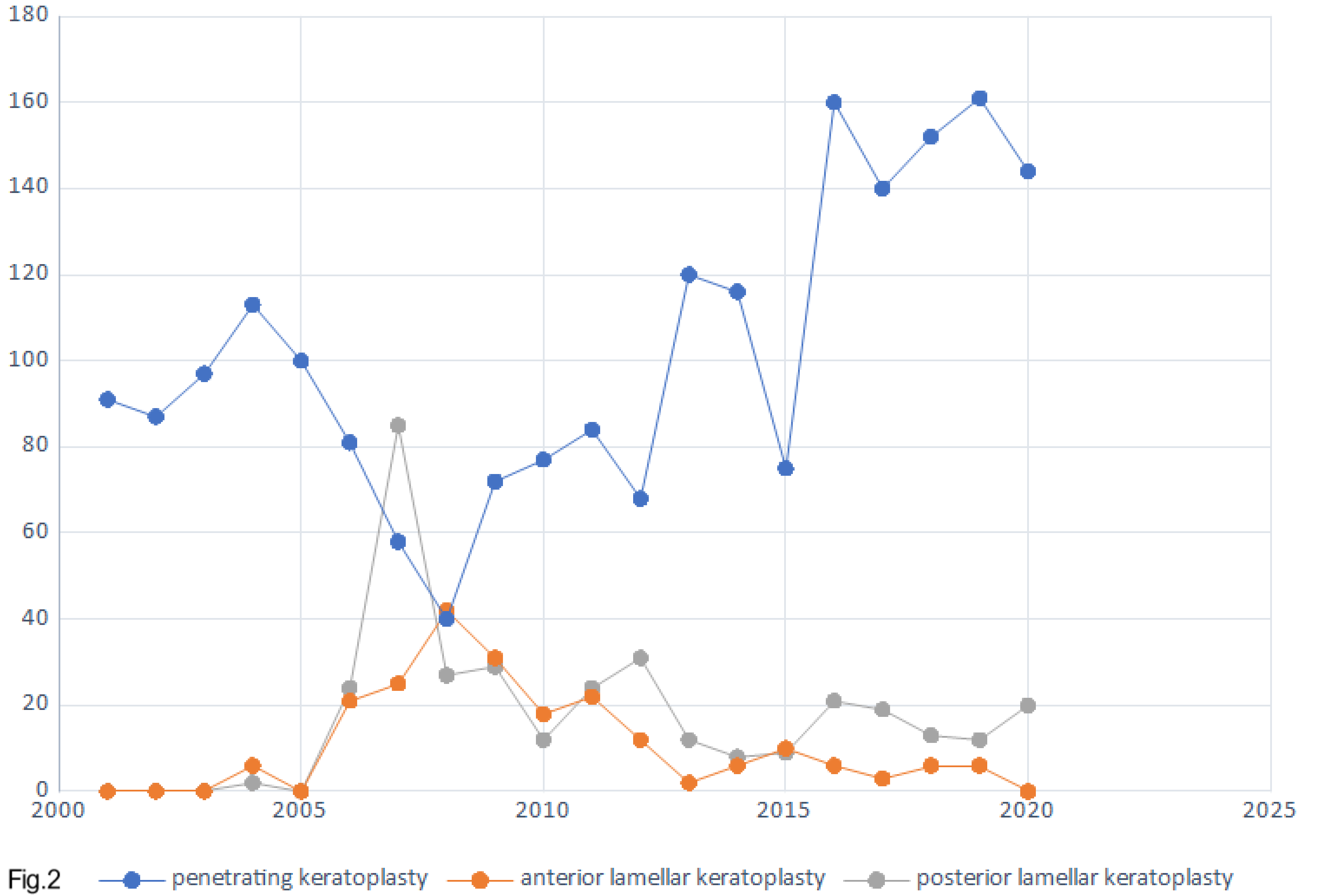
180
160
140
120
100
80
60
40
20
0
2000
2005
2010
2015
2020
2025
penetrating keratoplasty
anterior lamellar keratoplasty
posterior lamellar keratoplasty

Fig.2

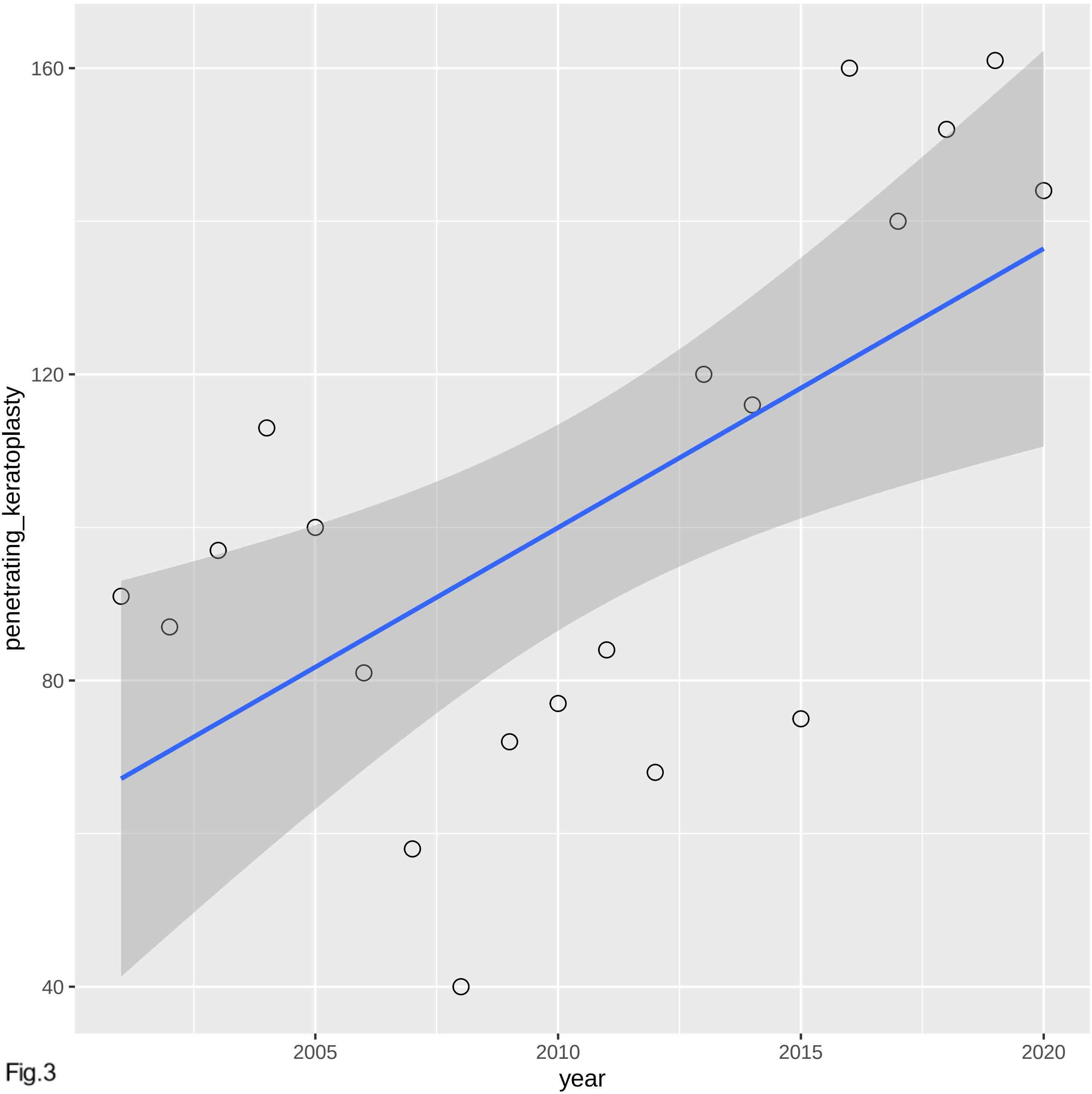

160
120
80
40
penetrating_keratoplasty
2005
2010
2015
2020
year

Fig.3

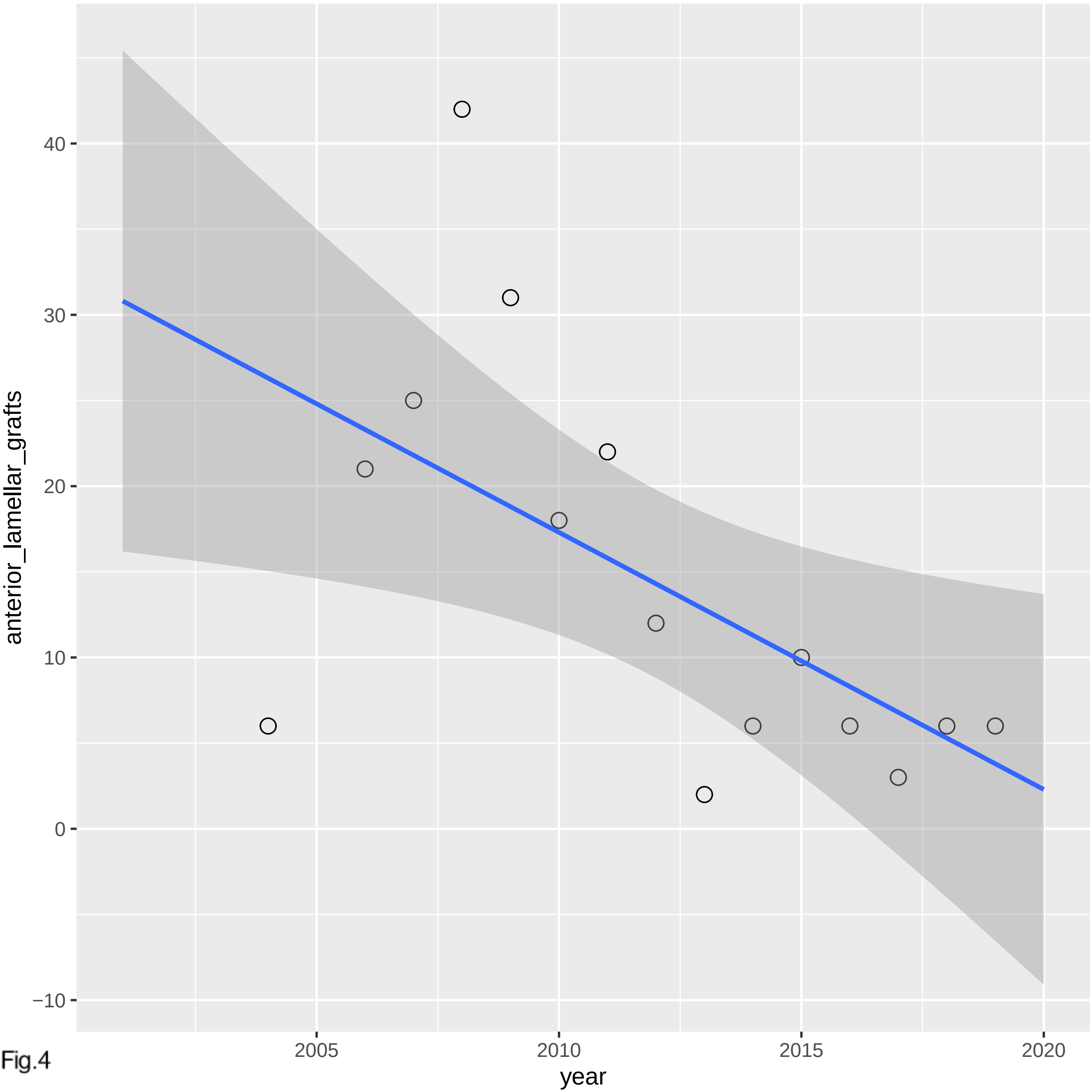
anterior_lamellar_grafts
40
30
20
10
0
−10
2005
2010
2015
2020
year

Fig.4

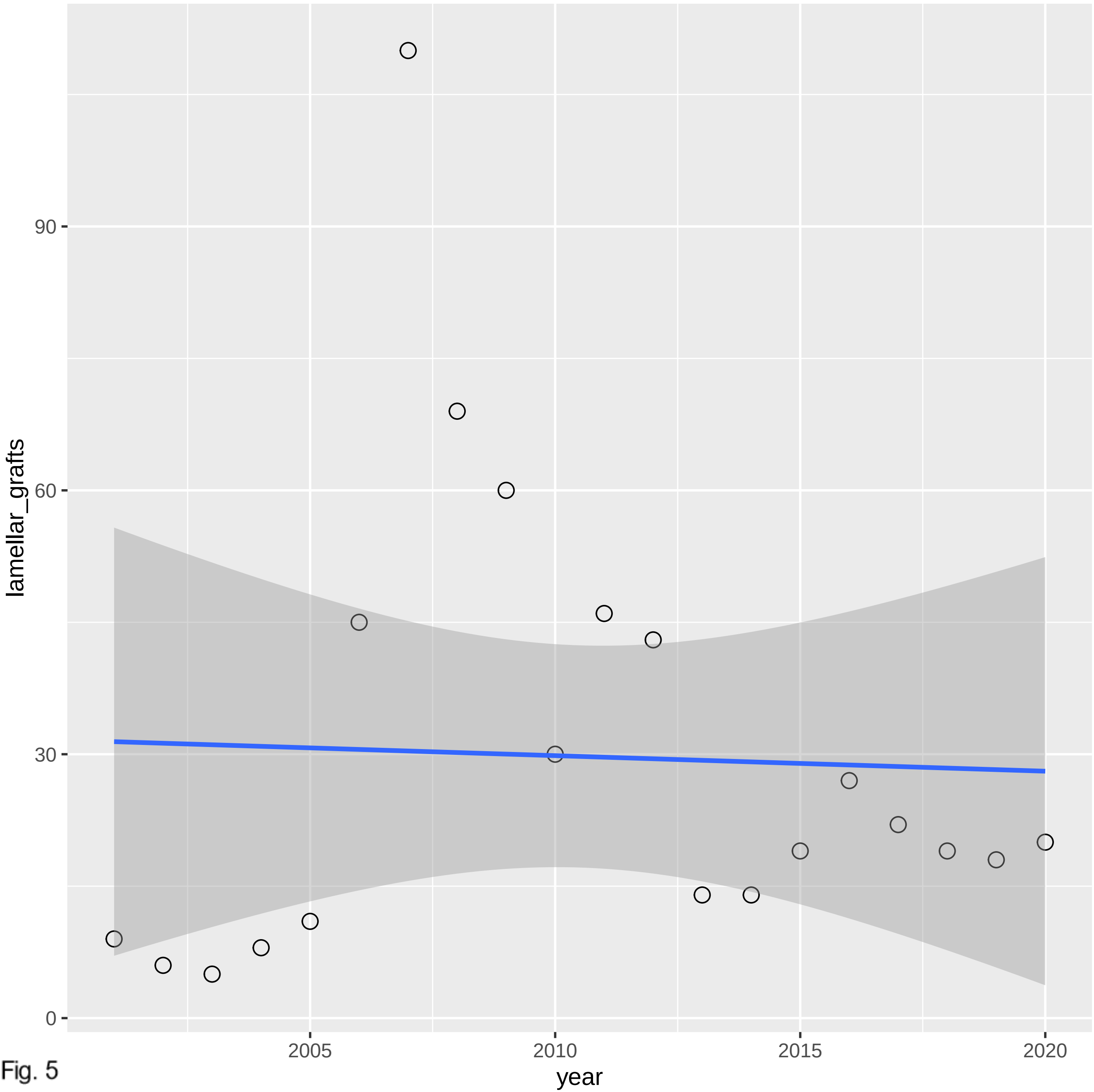

Fig. 5

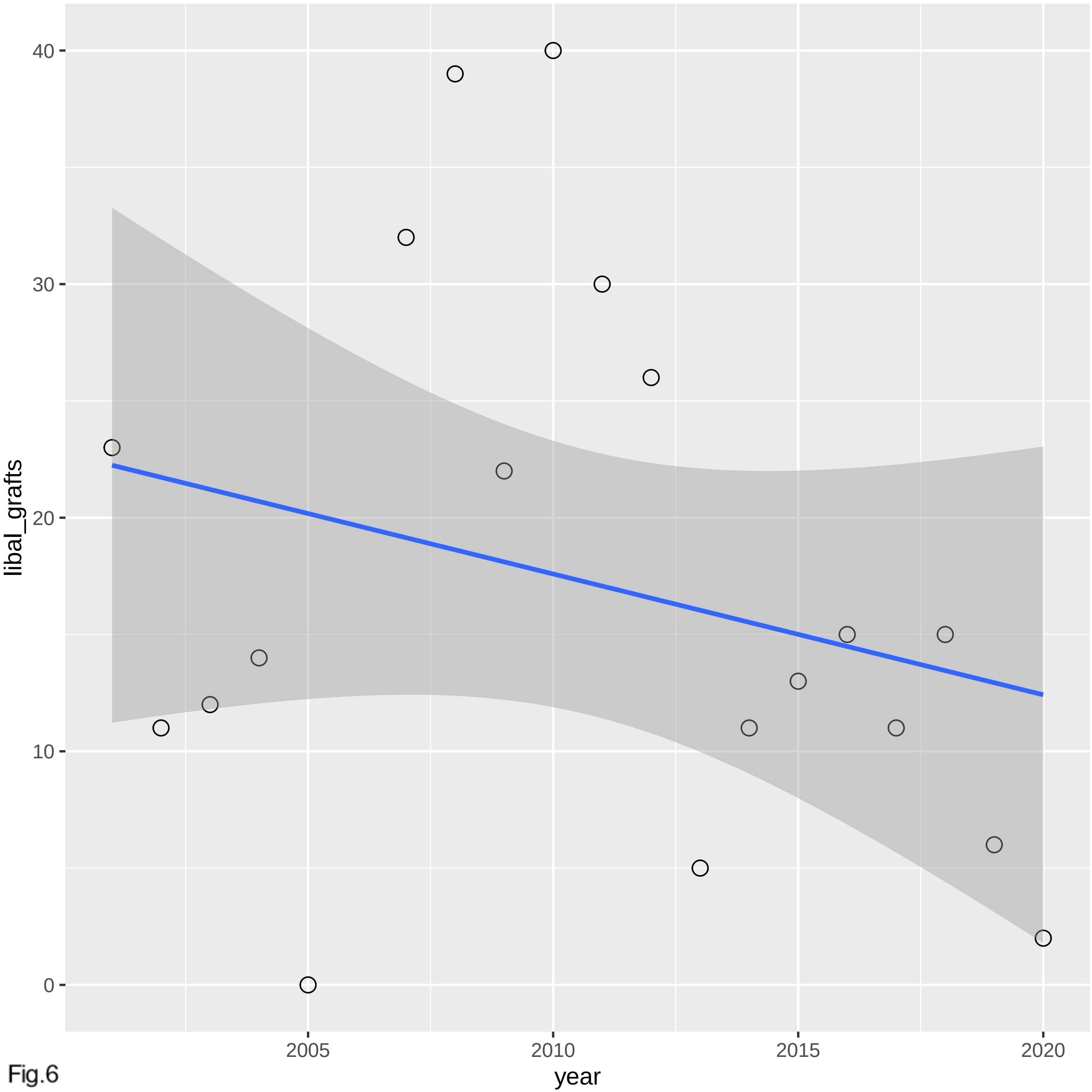

Fig.6

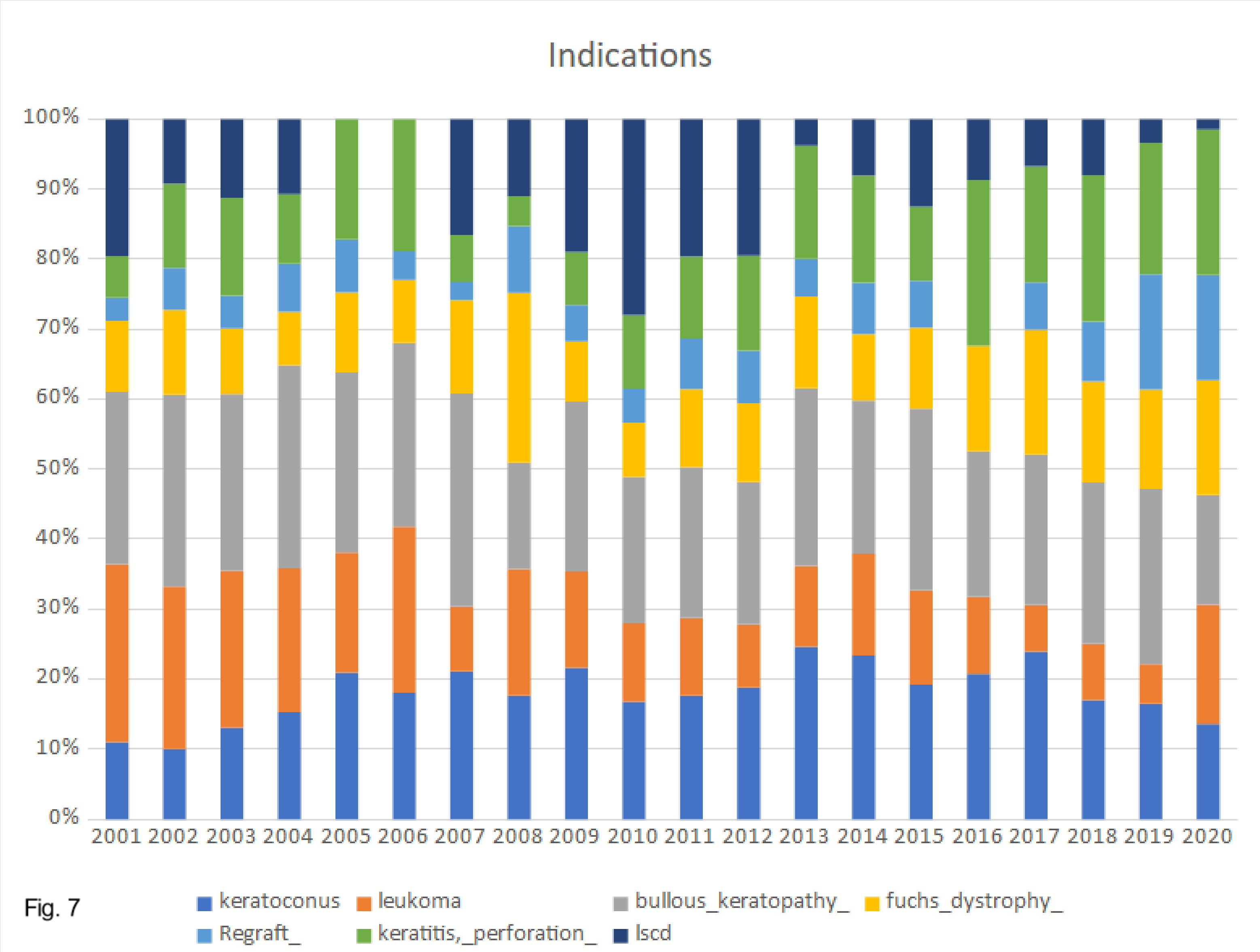

Indications
100%
90%
80%
70%
60%
50%
40%
30%
20%
10%
0%
2001 2002 2003 2004 2005 2006 2007 2008 2009 2010 2011 2012 2013 2014 2015 2016 2017 2018 2019 2020
keratoconus
leukoma
bullous_keratopathy_
fuchs_dystrophy_
Regraft_
keratitis,_perforation_
lscd

Fig. 7

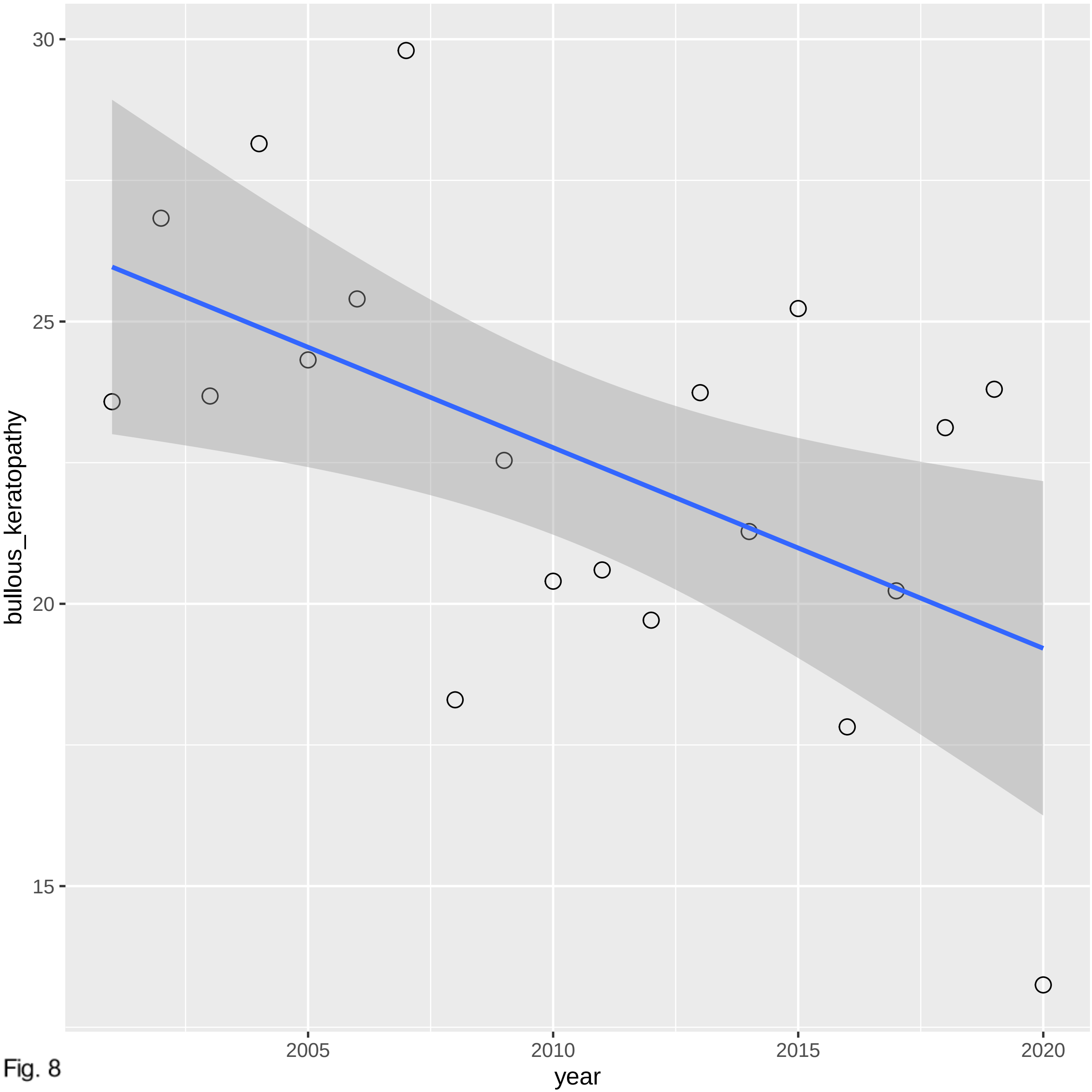

Fig. 8

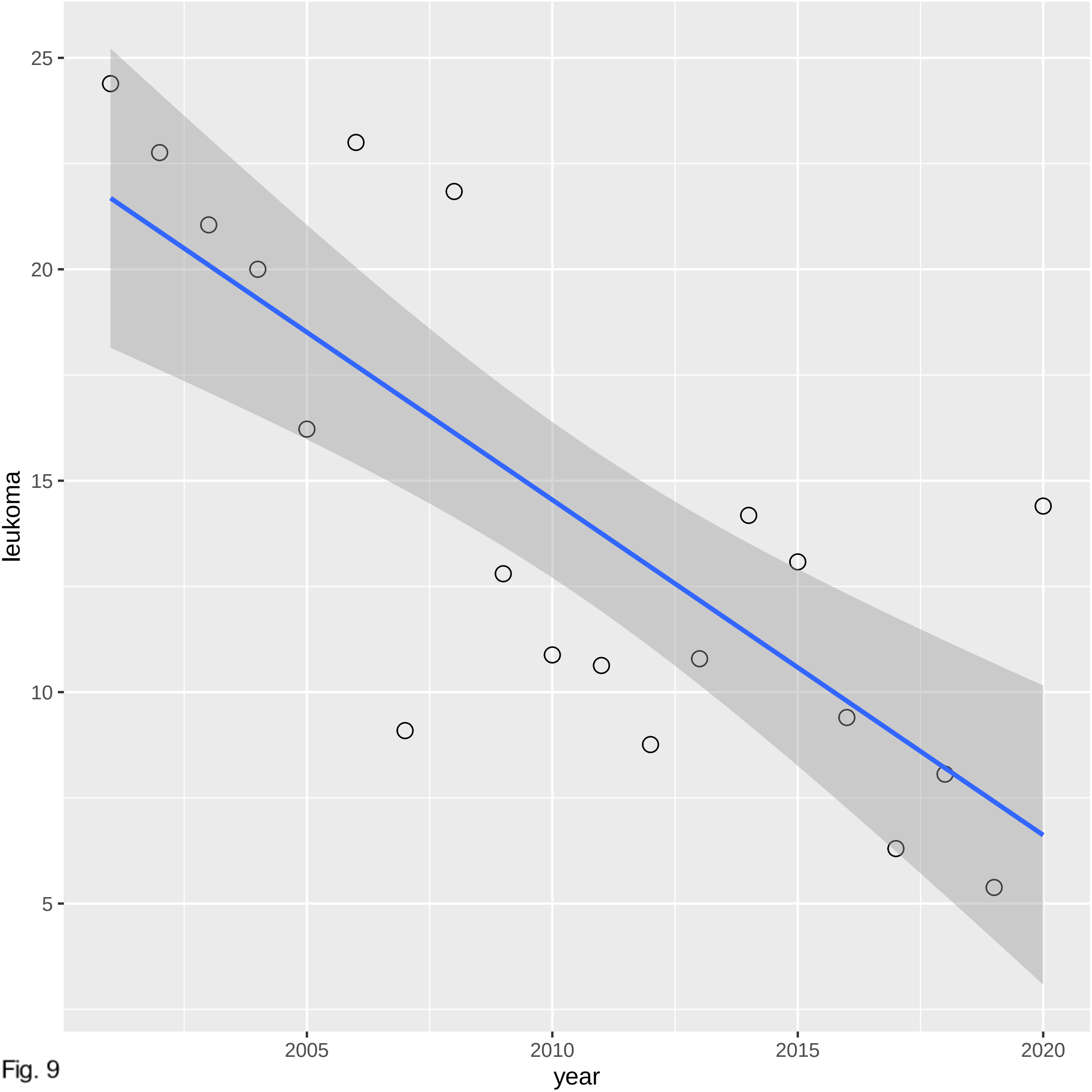

Fig. 9

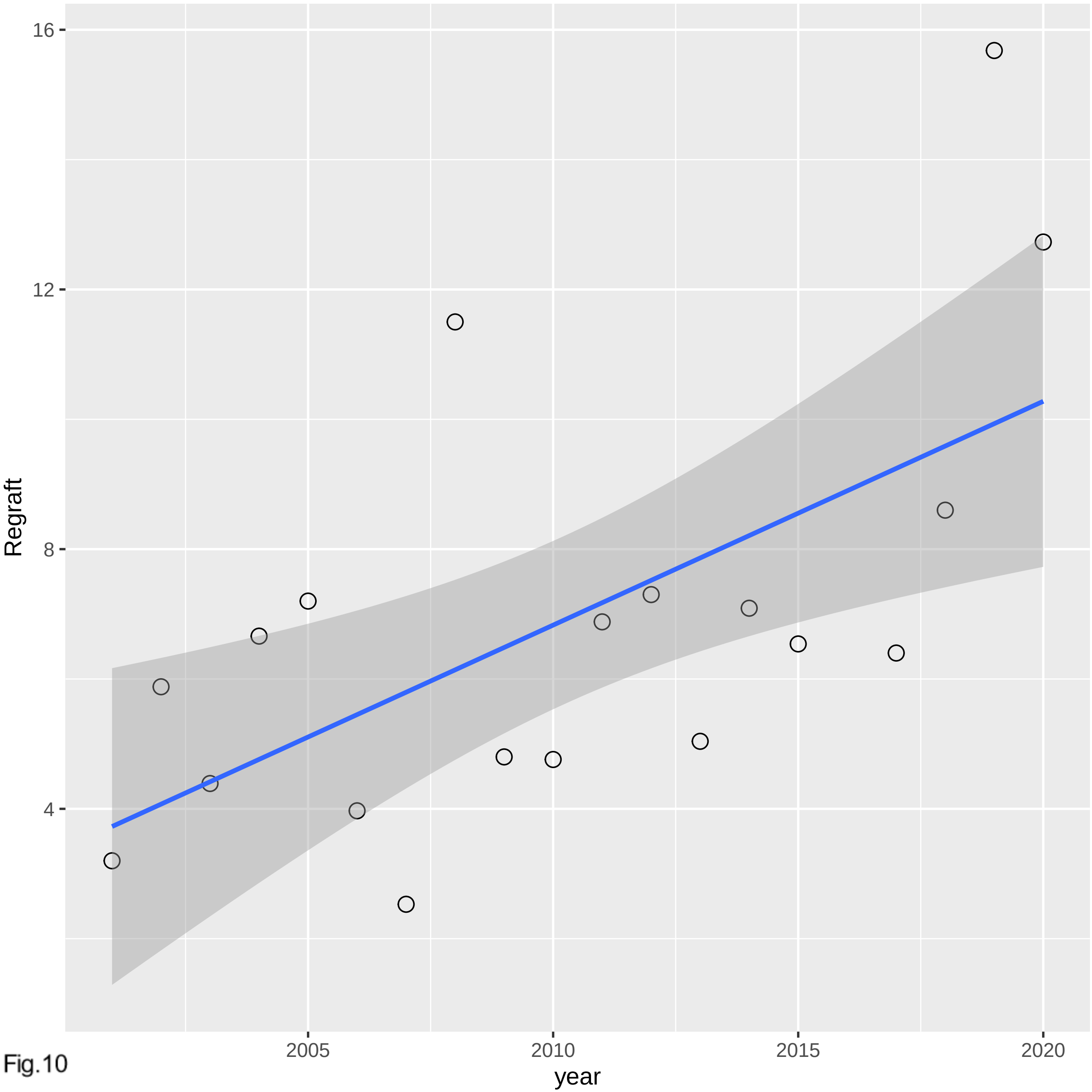

Fig.10

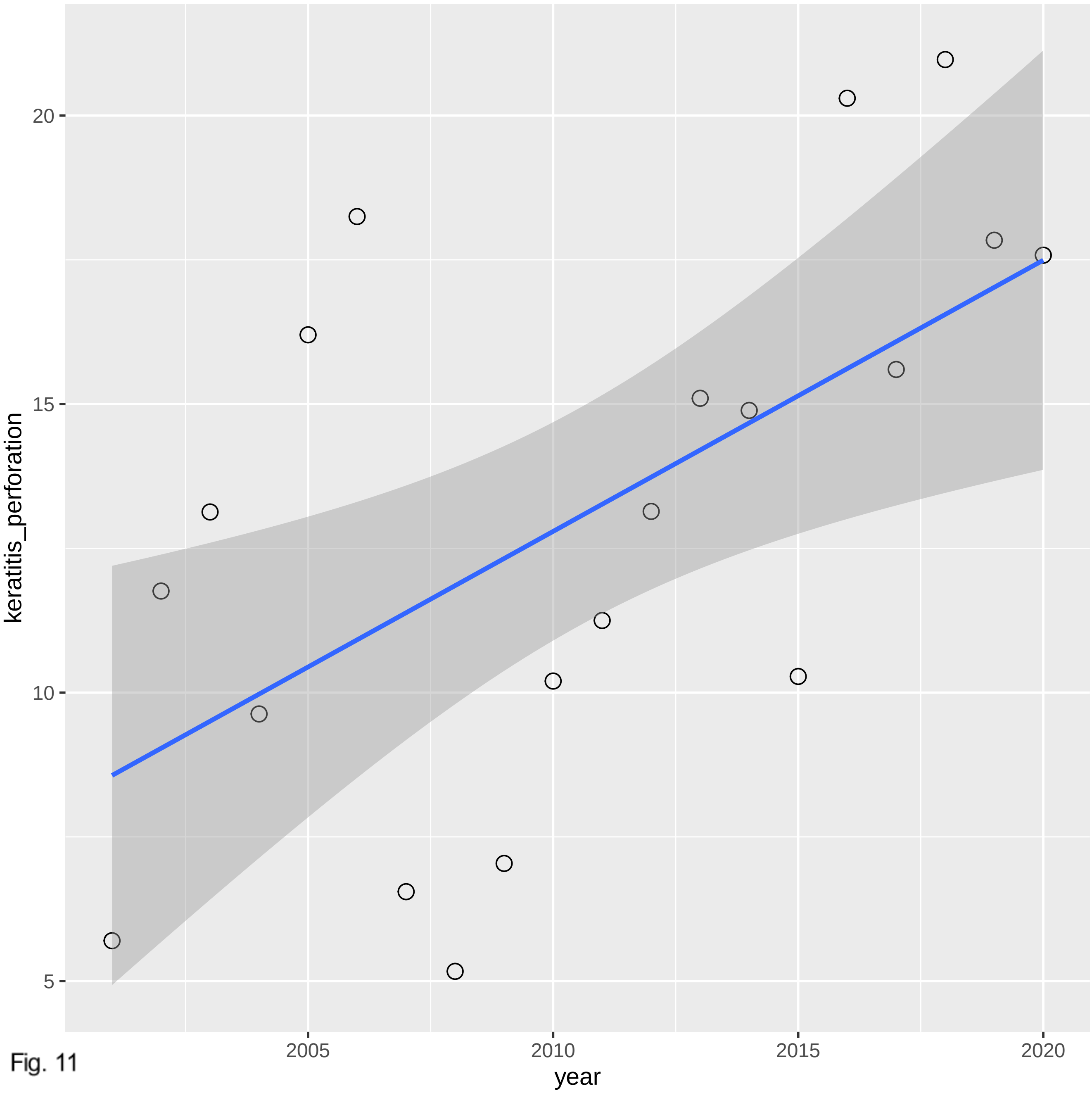

Fig. 11

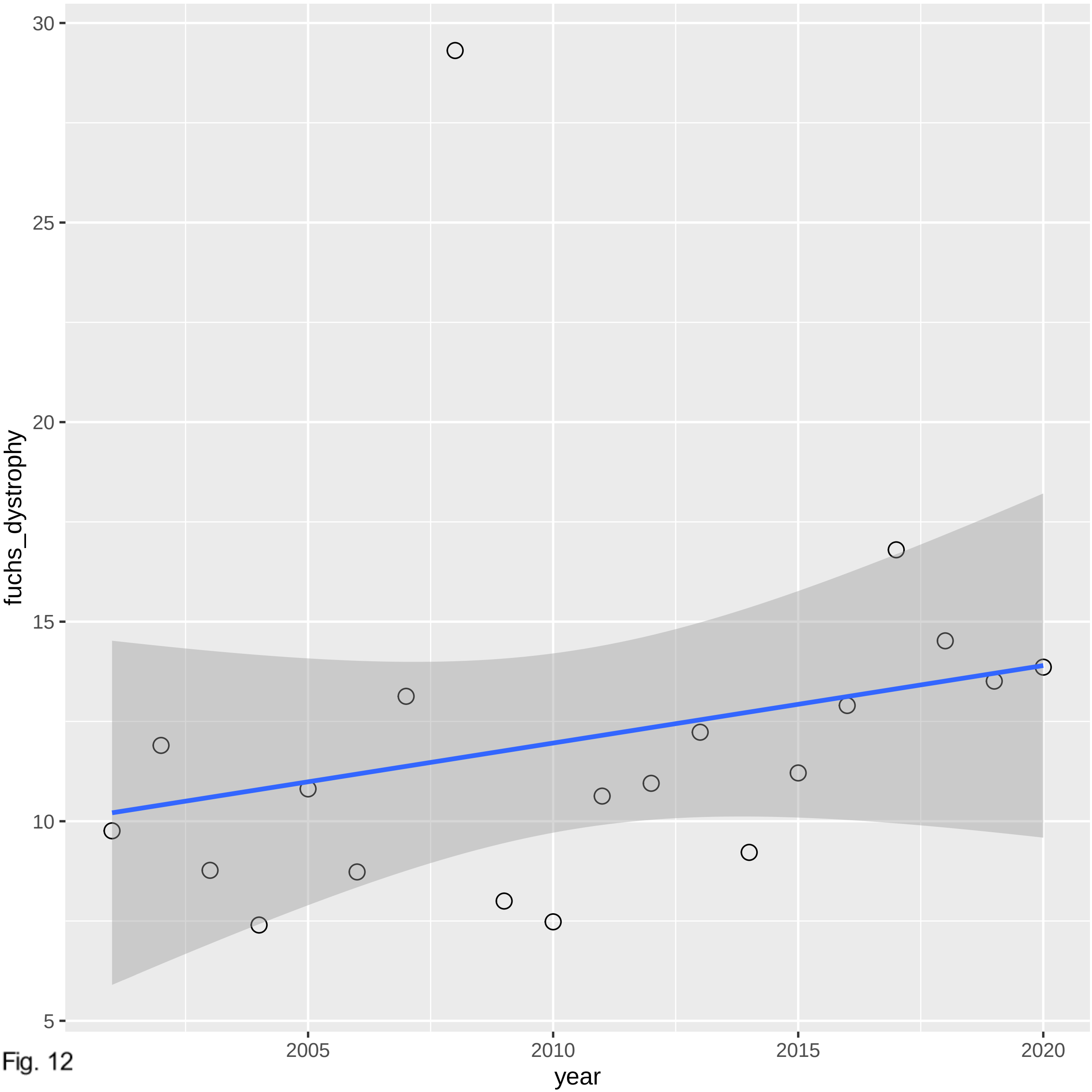

Fig. 12

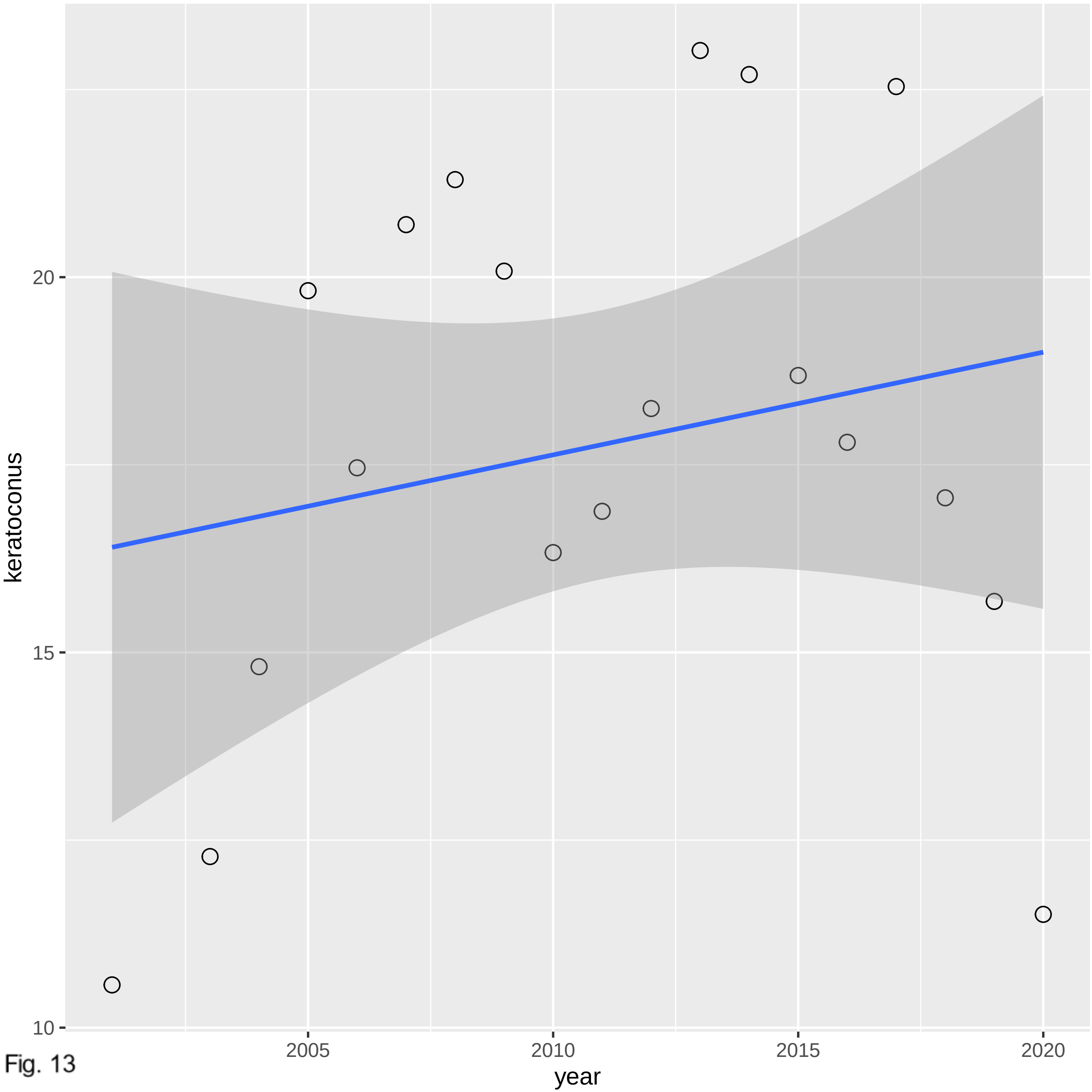

Fig. 13
